# Portable Bacterial Cellulose-Based Fluorescent Sensor for Rapid and Sensitive Detection of Copper in Food and Environmental Samples

**DOI:** 10.3390/molecules30173633

**Published:** 2025-09-05

**Authors:** Hongyuan Zhang, Qian Zhang, Xiaona Ji, Bing Han, Jieqiong Wang, Ce Han

**Affiliations:** 1 School of Science, Changchun Institute of Technology, 395 Kuanping Road, Changchun 130012, China; 2208411109@stu.ccit.edu.cn (Q.Z.); lx_jxn@ccit.edu.cn (X.J.); hanbing626@hotmail.com (B.H.); 2 School of Materials Science and Engineering, Changchun University, 6543, Weixing Road, Changchun 130022, China; 3 State Key Laboratory of Electroanalytical Chemistry, Changchun Institute of Applied Chemistry, Chinese Academy of Sciences, Changchun 130022, China

**Keywords:** carbon dots, fluorescent sensors, bacterial cellulose

## Abstract

Copper ions (Cu^2+^), indispensable in physiological processes yet toxic at elevated concentrations, require sensitive on-site monitoring. Here, a portable fluorescent sensing film (Y-CDs@BCM) was fabricated by anchoring yellow-emitting carbon dots (Y-CDs) into bacterial cellulose films, which enables rapid and sensitive detection of Cu^2+^ in complex real-world samples. The yellow fluorescent carbon dots (Y-CDs) were synthesized with the aid of o-phenylenediamine and 1-octyl-3-methylimidazolium tetrafluoroborate as precursors, exhibiting excellent fluorescence stability. The fluorescence of Y-CDs was selectively quenched by Cu^2+^ via the inner filter effect (IFE), allowing quantitative analysis with superior sensitivity compared to existing methods. By adding bacterial cellulose (BC) as a solid support, aggregation-induced fluorescence quenching was effectively reduced, and sensor robustness and portability were improved. Through smartphone-based colorimetric analysis, the Y-CDs@BCM sensor enabled rapid, visual interpretation of Cu^2+^ detection (within 1 min). Furthermore, cell viability and in vivo assays confirmed the biocompatibility of Y-CDs, indicating their suitability for biological imaging. This work presents an environmentally friendly, reliable, and practical method for on-site Cu^2+^ monitoring, emphasizing its broad application potential in food safety control and environmental analysis.

## 1. Introduction

The essential trace element copper ions (Cu^2+^) play an important role in regulating a variety of physiological and metabolic processes, including neural transmission, redox reactions, and enzymatic activity regulation [1,2]. However, dysregulated copper levels—particularly excessive accumulation—can trigger oxidative stress, liver and kidney damage, and various neurodegenerative conditions [3]. Accordingly, the accurate and sensitive detection of Cu^2+^ was of great importance for safeguarding both human health and food safety. As industrial pollution and agricultural discharges have increased, the accumulation of Cu^2+^ in aquatic environments has caused escalating environmental concerns, which underscores the urgency for reliable monitoring techniques in order to facilitate early warnings and effective remediation. For the detection of copper ions, the primary methods were atomic absorption spectroscopy (AAS) [4], inductively coupled plasma mass spectrometry (ICP-MS) [5], and electrochemical analysis [6]. Although these techniques provide high sensitivity and selectivity, they usually require expensive instruments, complex sample preparation procedures, and skilled personnel, making them unsuitable for portable, on-site, and rapid analysis. Hence, the development of a simple, fast, sensitive, and robust strategy for Cu^2+^ detection that is compatible with complex sample matrices remains a critical challenge in the field of analytical chemistry.

In recent years, carbon dots (CDs), an emerging class of zero-dimensional (0D) carbon-based nanomaterials, have attracted attention owing to their excellent fluorescence property, good biocompatibility, low toxicity, casual surface functionalization, and eco-friendly synthetic routes [7]. Due to these properties, their application in food safety, environmental monitoring, and biosensing, specifically for the detection of metal ions, has gained popularity [8]. Due to nitrogen and oxygen functional groups on the surface of CDs, they have improved photostability. Furthermore, they offer excellent coordination sites for metal ions [9]. This facilitates metal-induced fluorescence quenching with high sensitivity and selectivity. Nonetheless, traditional solution-phase CDs are often susceptible to aggregation-caused quenching (ACQ) and matrix interference in detection, which compromise their stability and reproducibility [10]. Moreover, their distribution in the liquid media makes recovery and post-processing of treatment more difficult, restricting on-site analysis and real-sample detection. As solid-phase platforms for the immobilization of CDs are developed, we may overcome these challenges. By preventing aggregation in aqueous environments, such platforms can improve the sensitivity and stability of the sensing system.

Bacterial cellulose (BC) has recently gained popularity due to its low-cost, sustainable, and biodegradable nature—a good alternative to non-degradable synthetic polymers. Due to its three-dimensional nanofibrous network, mechanical strength, hydrophilicity, and biocompatibility, BC has been successfully used in biomedicine, sensing materials, and environmental monitoring [11,12]. Wei et al. reported the self-assembly of nitrogen-doped carbon dots on BC membranes for Fe^3+^ ions detection [13]. Duan et al. created a biomimetic composite from BC-based carbon nanofibres (BCCFs) and silver oxide (AgO) for H_2_O_2_ vapour sensing [14]. Yang et al. fabricated multi-color fluorescent BC membranes for the evaluation of acidity in historical paper artifacts [15]. The studies have proven that BC is highly crystalline with excellent mechanical strength and a fine 3D network structure. Serving as a functional template, BC has shown significant potential in optical sensing platforms. Moreover, the high concentration of hydroxyl groups in BC can facilitate firm hydrogen bonding with CDs to promote their stable immobilisation and restrain their aggregation in aqueous media [16,17]. Therefore, the development of a portable fluorescent sensor based on BC as the supporting matrix and CDs as the fluorescent probe not only facilitates uniform dispersion and provides a favourable microenvironment for detection, but also offers advantages such as user-friendly handling, intuitive visual readout, and simplified sample preparation, making it highly suitable for field applications.

In this study, a portable fluorescent sensing membrane (Y-CDs@BCM) was fabricated for the detection of Cu^2+^ in pig liver and environmental water samples. The yellow-emissive carbon dots (Y-CDs), serving as the sensing unit, were synthesized using o-phenylenediamine (OPD) as the nitrogen source and 1-octyl-3-methylimidazolium tetrafluoroborate ([OMIM]BF_4_) as the boron and fluorine sources. Notably, due to the inner filter effect (IFE), the fluorescence emission of Y-CDs at 570 nm showed a quenching behaviour toward Cu^2+^ with concentration dependence, along with good sensitivity and selectivity. With the smartphone-based color recognition tool, the Y-CDs@BCM membrane was used to analyze Cu^2+^ visually and conveniently. The limit of detection achieved was lower than most previously reported approaches (7.758 nM). The fluorometric and visual analysis results of the sensor were successfully used to determine the Cu^2+^ in pig liver, serum, and environmental water samples with an acceptable recovery rate. Therefore, the developed solid-state sensing platform based on Y-CDs@BCM not only holds significant theoretical value for the design of portable Cu^2+^ probes but also offers a green and feasible strategy for the on-site, visual detection of Cu^2+^ in complex biological and environmental matrices.

## 2. Results

### 2.1. Synthesis and Characterization of Y-CDs

**Figure 1:**
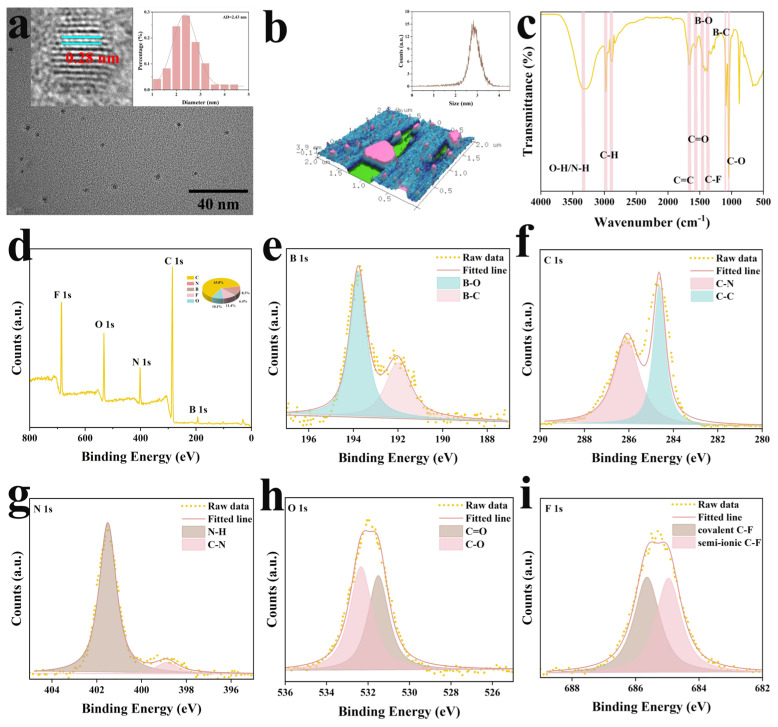
(**a**) TEM image of Y-CDs (inset: corresponding HR-TEM image and size histogram distribution); (**b**) AFM image of Y-CDs (inset: corresponding *Z*-axis height histogram distribution); (**c**) FT-IR spectrum and (**d**) full XPS spectrum; (**e**) high-resolution spectra of B 1s, (**f**) C 1s, (**g**) N 1s, (**h**) O 1s, and (**i**) F 1s of Y-CDs.

Y-CDs exhibited uniformly sized, well-dispersed spherical particles with an average diameter of 2.43 nm and a particle size distribution ranging from 1.2 to 4.4 nm. Statistical analysis indicated that approximately 71.9% of the particles fell within the diameter range of 2 to 2.8 nm, and the particle size distribution presented a normal distribution. Clear lattice fringes with a spacing of 0.28 nm, typical for graphitic carbon, were visible in the Y-CDs (Figure 1a, inset). Furthermore, atomic force microscopy (AFM) was employed to estimate the size and height of these Y-CDs (Figure 1b). The AFM analysis confirmed that the Y-CDs were spherical with a narrow size distribution, exhibiting an average vertical (*Z*-axis) dimension ranging from 0 to 4.5 nm (Figure 1b, inset), consistent with the TEM observations [9]. This phenomenon is attributed to the high temperature and prolonged synthesis duration during preparation, which promoted the transformation of the amorphous core into graphitic CDs (Y-CDs). Compared to amorphous CDs, Y-CDs are smaller and possess fewer surface functional groups, thus contributing to their superior stability during detection [18].

In general, the D vibration band observed in Raman spectroscopy is associated with structural disorder in the lattice of CDs, while the G band relates to planar vibrations of sp^2^-hybridized carbon atoms and phonon lattice vibrations [19]. The relatively high I_D_/I_G_ value (0.97) confirmed the high degree of graphitization in the Y-CDs (Figure S1). Fourier transform infrared spectroscopy (FT-IR) revealed a broad peak at 3316 cm^−1^, attributed to the stretching vibrations of O-H and N-H [20]. Sharp peaks observed at 2970 cm^−1^ and 2872 cm^−1^ corresponded to C-H stretching vibrations in CH_3_ and CH_2_ groups, respectively. Additionally, two prominent absorption peaks appeared at 1667 cm^−1^ and 1044 cm^−1^, corresponding to the stretching vibrations of C=O and C-O groups [21,22], respectively, alongside the bending vibration of C=C at 1560 cm^−1^. Furthermore, characteristic peaks at 1449 cm^−1^ and 1088 cm^−1^ were assigned to the asymmetric stretching vibration of B-O in the B-O-C structure and the stretching vibration of B-C in the B-C-O framework [23], respectively. An in-plane bending vibration of C-F was also observed at 1373 cm^−1^ (Figure 1c) [24].

X-ray photoelectron spectroscopy (XPS) further confirmed the electron binding energy and surface elemental composition of the Y-CDs. The full XPS survey spectrum is shown in Figure 1d. Peaks located at 193.52 eV, 285.09 eV, 402.00 eV, 532.06 eV, and 685.64 eV were attributed to B 1s, C 1s, N 1s, O 1s, and F 1s, respectively, with corresponding elemental contents of 6.38%, 63.78%, 8.31%, 10.11%, and 11.42%, respectively. The B 1s spectrum of Y-CDs was deconvoluted into two distinct peaks at 193.79 eV and 192.03 eV, corresponding to B-O and B-C bonds, respectively (Figure 1e) [25]. In the C 1s spectrum, peaks at 286.09 eV and 284.64 eV were attributed to C-O and C=O structures [26], respectively (Figure 1f). The N 1s spectrum was resolved into two peaks, confirming the presence of two nitrogen states: N-H (399.4 eV), which belonged to pyrrolic nitrogen, and C-N (399.8 eV), corresponding to pyridinic nitrogen at the carbon skeleton edges (Figure 1g) [27]. In the O 1s spectrum, peaks at 532.31 eV and 531.47 eV were assigned to C=O and C-O structures, respectively (Figure 1h) [28]. Meanwhile, peaks at 685.63 eV and 684.97 eV in the F 1s spectrum were attributed to semi-ionic and covalent C-F bonds, respectively (Figure 1i) [29].

In summary, FT-IR and XPS analyses were in good agreement, confirming the successful doping of N, F, and B atoms into the Y-CDs. The presence of these neutral polar functional groups enabled excellent dispersion of Y-CDs in aqueous solutions, thus enhancing their fluorescence properties.

### 2.2. Optical Properties of Y-CDs

**Figure 2:**
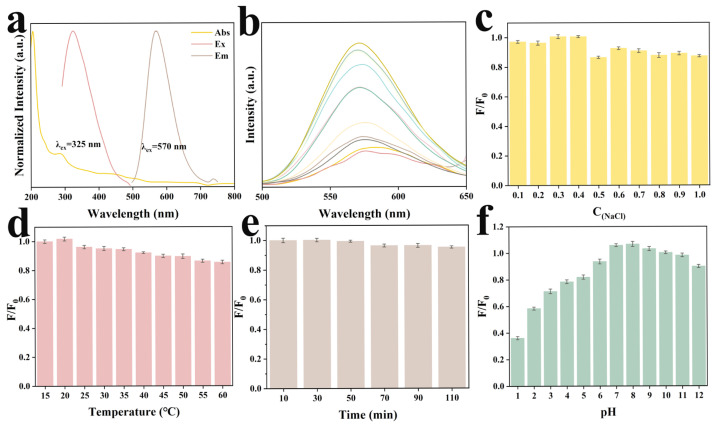
(**a**) Absorption, fluorescence excitation, and emission spectra of Y-CDs. (**b**) Fluorescence emission spectra of Y-CDs solutions at different excitation wavelengths. (**c**) Ionic strength stability test of Y-CDs. (**d**) Temperature stability test of Y-CDs. (**e**) Photobleaching time stability test of Y-CDs. (**f**) pH stability test of Y-CDs (error bars represented the standard deviations, which were calculated from three sets of data for the same sample.).

The optical properties of Y-CDs were systematically investigated through UV–visible absorption and fluorescence spectroscopy. As anticipated, two prominent absorption peaks were observed. The stronger absorption band at 238.44 nm was attributed to the typical π–π* transition of the sp^2^ carbon core domain, with its altered shape ascribed to the introduction of boron dopants. The absorption peak at 287.27 nm corresponded to the n–π* transition from lone pair electrons of surface functional groups to the π* orbitals of the sp^2^ domain [30]. Upon excitation at 325 nm, the Y-CDs exhibited a maximum emission at 570 nm (Figure 2a). The emission spectra recorded at excitation wavelengths ranging from 300 to 400 nm revealed no obvious red shift in the emission maximum, confirming the excitation-independent fluorescence behavior of the Y-CDs (Figure 2b). Using transient fluorescence spectroscopy, we determined that the Y-CDs had a fluorescence quantum yield of 19.41%.

We evaluated the fluorescence stability of the Y-CDs comprehensively to determine whether the sensor can be applied to complex sample matrices. It was found that Y-CDs maintained a fluorescence intensity above 87% even at NaCl concentrations of 1.0 M, suggesting robust ionic tolerance (Figure 2c). The high temperature resistance was investigated by heating the aqueous dispersion of Y-CDs. The results showed that Y-CDs could still maintain a high fluorescence emission efficiency under higher temperature conditions (60 °C) (Figure 2d). Considering the importance of photostability for sensor applications, we examined photobleaching resistance. Under continuous 365 nm UV irradiation, fluorescence intensity showed negligible degradation over a period of 110 min (Figure 2e). The pH-dependent photoluminescence behavior of Y-CDs was investigated in light of previous studies indicating that increasing pH can result in the deprotonation of surface functional groups on CDs [31]. According to the results, fluorescence intensity significantly increased from pH 1 to pH 7, with no significant decrease observed between pH 8 and 12. As a result of enhanced vibrational coupling, Y-CDs may be more rigid and lose less energy during charge transfer due to enhanced vibrational coupling, resulting in a greater number of excited electrons releasing photons during relaxation, resulting in greater fluorescence (Figure 2f).

### 2.3. Quenching Mechanism

**Figure 3:**
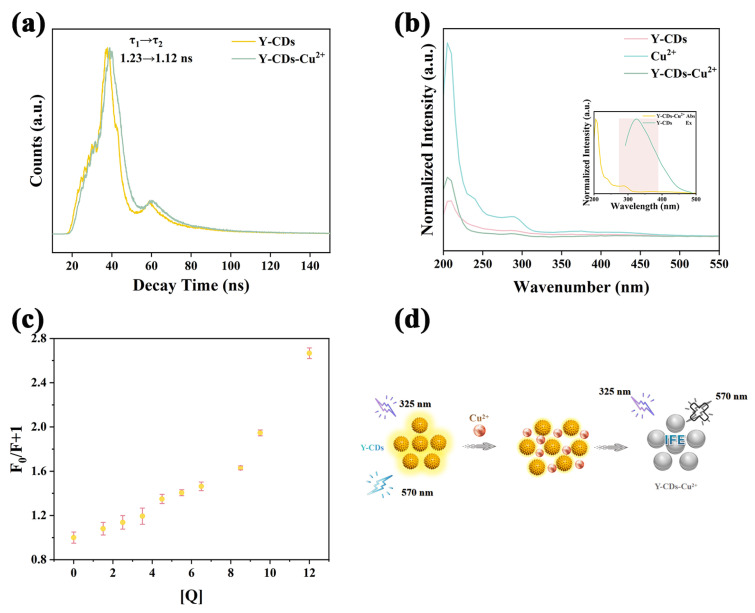
(**a**) Fluorescence lifetime of Y-CDs in the presence and absence of Cu^2+^. (**b**) UV-visible absorption spectra of Y-CD, Cu^2+^, and the Y-CDs-Cu^2+^ system (inset: UV absorption of Cu^2+^ and fluorescence excitation spectrum of Y-CDs). (**c**) Stern–Volmer fitting curve. (**d**) Schematic diagram of the quenching mechanism.

Generally, quenching mechanisms could be categorized as static quenching, dynamic quenching, fluorescence resonance energy transfer (FRET), photoinduced electron transfer (PET), and the inner filter effect (IFE). To elucidate the interaction mechanism between Y-CDs and Cu^2+^, the fluorescence lifetime of Y-CDs was measured in the presence and absence of Cu^2+^. The results showed that the fluorescence lifetime remained essentially unchanged under both conditions (τ_1_ = 1.23 ns, τ_2_ = 1.12 ns, τ_1_/τ_2_ = 1.10), thereby excluding dynamic quenching and FRET as possible mechanisms (Figure 3a). Furthermore, upon addition of Cu^2+^, the UV-visible absorption spectrum of the Y-CDs-Cu^2+^ system exhibited no significant red or blue shifts at 238.44 nm and 287.27 nm, indicating that the formation of a non-fluorescent ground state complex was unlikely (Figure 3b). Notably, there was significant spectral overlap between the characteristic UV absorption band of Cu^2+^ and the excitation spectrum of Y-CDs (Figure 3b, inset). The results of these studies led us to conclude that the quenching of Y-CDs by Cu^2+^ was primarily controlled by the inner filter effect (IFE). In order to verify this hypothesis quantitatively, we employed the Stern–Volmer equation (Equation (1)) for analysis.

(1) $$\frac{F_{0}}{F}=1+K_{SV}[Q]$$

Here, F_0_ represents the fluorescence intensity of Y-CDs in the absence of Cu^2+^; F denotes the fluorescence intensity after the addition of Cu^2+^; K_sv_ was the quenching constant between Y-CDs and Cu^2+^, and [Q] represents the Cu^2+^ concentration. The fitting results revealed that the relationship between F_0_/F and Cu^2+^ concentration conformed only to a polynomial fit. Moreover, the curve exhibited a pronounced upward curvature at higher concentrations, a deviation that aligns with the typical Stern–Volmer (SV) behavior under the influence of the inner filter effect (IFE) (Figure 3c) [32]. To further confirm this mechanism, the Parker equation (Equations (S1)–(S3)) was employed. With increasing Cu^2+^ concentration, the values of F_cor_/F_obsd_, E_obsd_, and E_cor_ progressively increased, thereby reinforcing the conclusion that IFE was the dominant quenching mechanism (Table S1). In summary, the concentration-dependent quenching behavior of Y-CDs by Cu^2+^ can be attributed to the inner filter effect (Figure 3d).

### 2.4. Selectivity and Sensitivity

**Figure 4:**
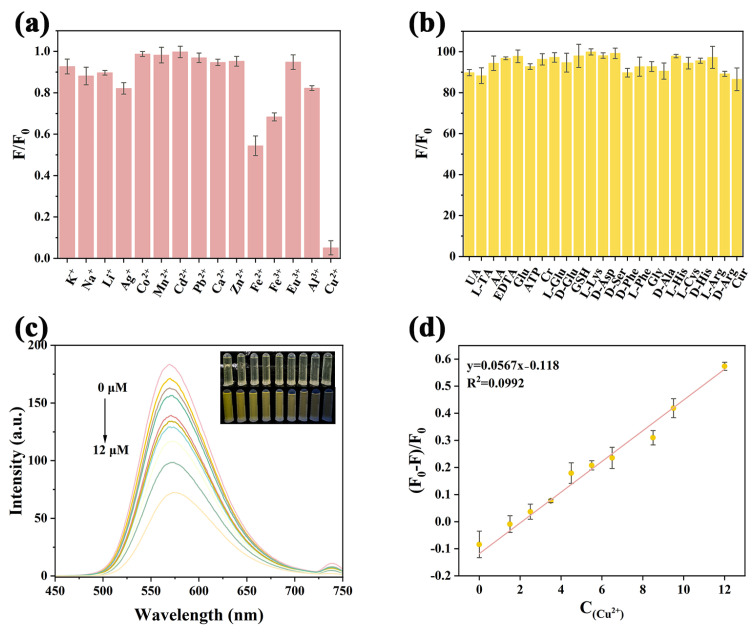
Selectivity of Y-CDs for various possible (**a**) metal ions and (**b**) small biomolecules. (**c**) The relationship between fluorescence intensity and different concentrations of Cu^2+^ (0–12 μM). (**d**) Linear plot of the (F_0_ − F)/F_0_ fluorescence intensity ratio in the presence of Cu^2+^ (error bars represent standard deviation, calculated from three sets of data for the same sample).

To ensure the selectivity of Y-CDs as a fluorescent probe for Cu2^+^ detection, their fluorescence response toward various ions (K^+^, Na^+^, Li^+^, Ag^+^, Co^2+^, Fe^2+^, Mn^2+^, Cd^2+^, Pb^2+^, Ca^2+^, Zn^2+^, Fe^3+^, Eu^3+^, Al^3+^, H_2_PO_4_^−^, Cl^−^, NO^3−^, F^−^, SO_4_^2−^, S_2_O_8_^2−^, and Cu^2+^, 0.1 M) and biomolecules (UA, L-TA, AA, EDTA, Glu, ATP, Cr, L-Glu, D-Glu, GSH, L-Lys, D-Asp, D-Ser, D-Phe, L-Phe, Gly, D-Ala, L-His, L-Cys, D-His, L-Arg, D-Arg, and Cur, 0.1 M) was systematically evaluated Figure 4a,b and Figure S2). The results revealed that Y-CDs exhibited negligible fluorescence changes in the presence of all tested species except Cu^2+^, which caused a marked quenching effect (Figure 4c). We observed that high concentrations of Fe^3+^ and Fe^2+^ partially quench the fluorescence of Y-CDs. Previous studies have attributed this to the formation of non-fluorescent complexes [31]. In our detection system, due to the low metal ion concentration of the analyte, this phenomenon is effectively circumvented by the presence of the IFE. Moreover, the fluorescence intensity of Y-CDs showed a distinct concentration-dependent decrease with increasing Cu^2+^ concentrations (0–12 μM), exhibiting good linearity as described by the linear equation y = 0.0567x − 0.118 (Figure 4d). The limit of detection (LOD) was calculated as 7.758 nM using the equation 3σ/k, where σ represents the standard deviation of the fluorescence intensity from 11 blank samples and k is the slope of the calibration curve. Notably, Y-CDs responded to Cu^2+^ within just 10 s (Figure S3), consistent with the characteristic timescale of the inner filter effect (IFE), further supporting their potential as a rapid response sensor for Cu^2+^ detection. Compared to previously reported methods (Table S2), Y-CDs demonstrated superior sensitivity. Overall, this sensing strategy offers excellent selectivity, fast response, and high sensitivity for trace-level Cu^2+^ detection.

### 2.5. Actual Sample Analysis

As a key factor in the enzymatic reactions of cytochrome c oxidation, copper amine oxidases and dopamine β-hydroxylase in the human body, Cu^2+^ can be absorbed into the human body through the small intestine. However, the abnormal accumulation of Cu^2+^ in the environment and food chain can not only destroy the ecological balance, but also cause food safety problems and ecological risks. Pursuing this context, we evaluated our Y-CDs@BCM sensing platform by detecting Cu^2+^ across complex matrices such as porcine liver, human serum, and environmental water. To demonstrate the applicability of this method for routine use, pig liver, serum, and environmental water samples were selected as real-world samples. Three spiked samples were spiked at different concentrations to evaluate the accuracy of the proposed method (n = 3). As shown in Table 1, the recoveries of the spiked Cu^2+^ were within the 98.08–116.13% range, with relative standard deviations (RSDs) as low as 0.35–2.48%. Collectively, these results show that the Y-CDs platform affords robust analytical performance, enabling matrix-tolerant, on-site quantification of Cu^2+^ at μM levels below the required level to drinking water public health and food safety thresholds (e.g., U.S. EPA action level, 1.3 mg·L^−1^; WHO guideline, 2 mg·L^−1^). Thus, the sensor offers a practical strategy for routine Cu^2+^ monitoring in food and environmental samples.

### 2.6. Y-CDs@BCM Portable Fluorescence Sensor

**Figure 5:**
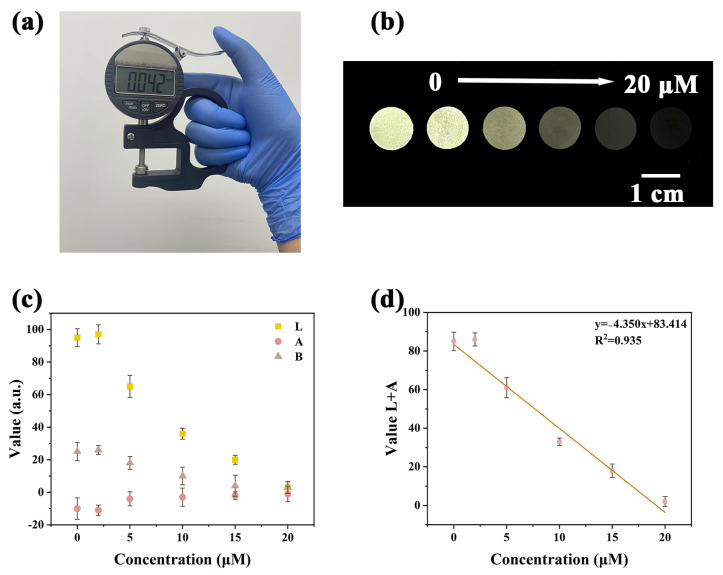
(**a**) Thickness measurement image of Y-CDs@BCM. (**b**) Fluorescence quenching images of the fluorescent film under 365 nm UV light after adding different concentrations of Cu^2+^. Linear relationships between (**c**) L A B values and (**d**) L + A values of Y-CDs@BCM reacted with Cu^2+^ concentration (0–20 μM).

Although portable fluorescent sensors for Cu^2+^ detection have been previously reported, highly sensitive, green, portable, visually readable, and environmentally adaptable sensing platforms remain scarce. Most composite sensors suffer from unstable responses and limited environmental sustainability. The porous structure, hydrophilicity, and high surface area of bacterial cellulose (BC) make it an ideal matrix for constructing composite materials. In this study, leveraging the excellent dispersibility and optical properties of Y-CDs, a Y-CDs@BCM fluorescent sensing film with a thickness of 0.042 mm was fabricated by mixing Y-CDs with BC via a vacuum filtration method (Figure 5a). The sensor exhibited a rapid response to Cu^2+^ concentrations ranging from 0–20 μM within 1 min. Furthermore, under 365 nm handheld UV light, the film displayed pronounced fluorescence quenching (Figure 5b). Using smartphone-based software (HUAWEI Mate XT, ColorDesk, V2.21), a good linear correlation was observed between the L and A color channels, with the relationship described by y = −4.350x + 83.414 (R^2^ = 0.935) (Figure 5c,d). Following previously reported procedures, the limit of detection (LOD) was calculated to be 24.266 nM using the formula 3σ/|k|, where σ represents the standard deviation of fluorescence intensities from 11 blank samples, and k is the slope of the calibration curve. The calculated LOD falls below established residue limits, confirming the feasibility of Y-CDs@BCM for on-site Cu^2+^ detection. In summary, the Y-CDs@BCM fluorescent sensing film demonstrates fast response, visual detectability, and high sensitivity, effectively addressing the limitations of traditional composite sensors in terms of response stability and environmental adaptability.

### 2.7. Cytotoxicity Test and Imaging

**Figure 6:**
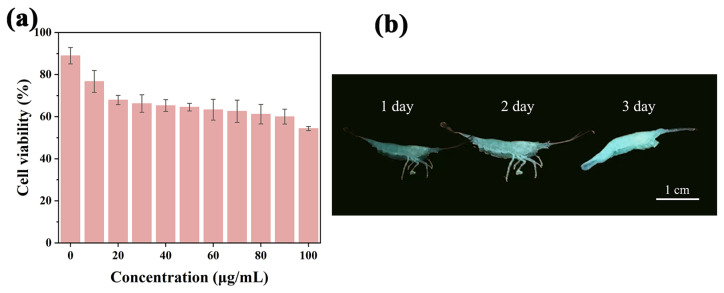
(**a**) The 4T1 cell viability with different concentrations of Y-CDs for 24 h. (**b**) Fluorescence images of freshwater shrimps at different incubation times.

To evaluate the biosafety of Y-CDs, we performed a standard MTT assay using 4T1 cells. After incubation with 400 μg mL^−1^ Y-CDs for 24 h, the cell viability remained above 85% (Figure 6a), demonstrating the low cytotoxicity of Y-CDs. Based on these findings, freshwater shrimp were selected to assess the feasibility of Y-CDs for in vivo fluorescence imaging. After incubation with Y-CDs in PBS buffer (pH 6.8) for 3 days, the live fluorescence of the shrimp was recorded using a smartphone under 365 nm UV light. The results showed that after 3 days of continuous incubation, there were no significant changes in the shrimp’s motility or appearance, and the shrimp exhibited yellow fluorescence (Figure 6b). This demonstrates that Y-CDs possess excellent fluorescence imaging performance and biosafety.

## 3. Materials and Methods

Procedures for handling all materials prior to testing and corresponding information for all tests are provided in the Supporting Information.

### 3.1. Reagents and Materials

O-phenylenediamine, 1-octyl-3-methylimidazolium tetrafluoroborate ([OMIN]BF_4_), and the interfering ions (KCl, NaCl, LiCl, CoCl_2_, PbCl_2_, CaCl_2_, ZnCl_2_, FeCl_2_, FeCl_3_, AlCl_3_, AgNO_3_ (ethanol-based silver nitrate solution), Eu(NO_3_)_3_·6H_2_O, Na_2_HPO_4_, K_2_HPO_4_, NaNO_3_, KNO_3_, NaF, KF, Na_2_SO_4_, K_2_S_2_O_8_, and biomolecules (uric acid, L-tartaric acid, ascorbic acid, ethylene diamine tetra-acetic acid, glutamic acid, L-glutamic acid, D-glutamic acid, glutathione, L-lysine, L-phenylalanine, D-phenylalanine, glycine, D-alanine, L-histidine, D-histidine, L-cysteine, L-arginine, D-arginine, D-serine, D-aspartic acid, adenosine triphosphate, creatinine, and curcumin) used in this experiment were purchased from Macklin (Shanghai, China). Bacterial cellulose dispersion (TL-008, BC) was purchased from Nanjing Tianlu Nanotechnology Co., Ltd. (Nanjing, China). Ultrapure water prepared by the Milli-Q Gradient Ultrapure Water System (Millipore, Burlington, MA, USA) was used during the experiment.

### 3.2. Instrument

The morphology and crystalline structure of the samples were examined using high-resolution transmission electron microscopy (JEOL-2100F, JEOL, Tokyo, Japan). UV–Vis absorption spectra were recorded with a spectrophotometer (Varian Cary 50, Agilent, Santa Clara, CA, USA). FT-IR spectra were obtained using a spectrometer (IS50, Thermo, Boston, MA, USA). Raman spectra were measured with a LABRAM HR Evolution system (LabRAM HR Evolutio, HORIBA, Irvine, CA, USA). X-ray photoelectron spectroscopy (XPS) measurements were performed using a monochromated Al Kα source on a diffractometer (ESCALAB Mk II, VG Scientific, Newburyport, MA, USA). Fluorescence excitation and emission spectra were collected using a spectrometer (LS-55, PerkinElmer, Shelton, CT, USA). During the test, the slit width of the excitation and emission spectra was set to 6 nm. Absolute photoluminescence quantum yields (Φ) were measured using a spectrofluorometer (QuantaMaster 8000, HORIBA, Irvine, CA, USA).

### 3.3. Preparation of Y-CDs

In total, 0.5 g of o-phenylenediamine (OPD) and 2 g of 1-octyl-3-methylimidazolium tetrafluoroborate ([OMIM]BF_4_) were dissolved in 20 mL of ethanol solution while stirring. Afterwards, the solution was put into a 50 mL Teflon-lined stainless-steel autoclave followed by hydrothermal treatment at 180 °C for 12 h. After natural cooling to room temperature, the resulting mixture was centrifuged at 13,400× *g* (r = 12 cm) for 15 min to remove insoluble residues. The supernatant was placed into a dialysis bag (1000 Da) and dialyzed for 24 h. Then, the solution was filtered again using a 0.22 μm filter and freeze-dried to obtain a deep brown Y-CDs powder, which was subsequently used for characterization experiments. Finally, the dialysate was freeze-dried to obtain yellow powder, which was re-dissolved in deionized water to achieve a stock solution of Y-CDs (1 mg/mL) for subsequent experiments and characterization of its morphology and chemical structure.

### 3.4. Stability of Y-CDs

To investigate the environmental stability of Y-CDs, 1 mg/mL aqueous solution was used for the following tests. Ionic strength was assessed by recording the fluorescence spectra of Y-CDs after the addition of NaCl (0.1–1.0 M). The pH stability of Y-CDs was evaluated by measuring their fluorescence intensity in a range between 1.0 and 11.0. To assess the thermal stability, 3 mL of Y-CDs (0.2 mg/mL) was heated in a thermostatic water bath for 10 min at different temperature points (30–50 °C). Photostability was examined by exposing the Y-CDs solution to 365 nm UV light for durations ranging from 10 to 120 min, followed by fluorescence intensity measurements. Additionally, fluorescence spectra were recorded using excitation wavelengths from 300 to 400 nm to study the excitation-dependent emission behavior. Finally, the absolute fluorescence quantum yield (Φ) was tested using a fluorescence spectrometer.

### 3.5. Preparation of Y-CDs@BCM Sensors

The Y-CDs@BCM composite membrane was prepared via vacuum filtration of a suspension containing bacterial cellulose (BC) and Y-CDs. In general, the nanocellulose suspension was diluted to 0.1 wt%, mixed with Y-CDs (5 mg/mL), and filtered under reduced pressure in a suction flask. Subsequent to filtration, the wet membrane was peeled from the filter and transferred to a PTFE-coated glass plate. The hydrogel membrane was dried at 100 °C for 6 h to yield the final Y-CDs@BCM film.

### 3.6. Detection of Cu^2+^

In total, 2.9 mL of different concentrations of Cu^2+^ solution (0–12 μM) was added to 100 μL of diluted Y-CDs (1 mg/mL). After reacting at room temperature for 30 s, the fluorescence emission spectrum of the Y-CDs-Cu^2+^ system was measured to quantitatively analyze Cu^2+^. For portable Cu^2+^ detection, Y-CDs@BCM was immersed in different concentrations of Cu^2+^ for 5 min. After that, Y-CDs@BCM was placed in a 365 nm dark box UV-visible light analyzer, and a smartphone (Huawei Mate XT, Huawei, Shenzhen, China) was placed in a fixed position. The fluorescence color was identified using color recognition software (ColorDesk, V2.21) and converted into LAB digital information. The purified Y-CDs solution and Y-CDs-Cu^2+^ (12 μM) system solution were prepared separately for the time-resolved emission spectrum of the tester to determine its fluorescence lifetime.

### 3.7. Pretreatment of Actual Samples

To evaluate the practical applicability of the Y-CDs-based fluorescent sensor, Cu^2+^ detection was performed in human serum, environmental water, and Pig liver samples using the standard addition method. Human serum samples were collected from the fingertip blood of volunteers and centrifuged at 13,400× *g* (r = 12 cm) for 10 min at 3 °C. The supernatant was then diluted 100-fold with ultrapure water. Environmental water samples were collected from South Lake (Changchun, China) and diluted 10-fold prior to use. Pig liver samples were purchased from a local farmers’ market and pretreated according to the Chinese national standard method (GB 5009.13-2017) [33]. Briefly, 1.0 g of pig liver was placed into a microwave digestion vessel, followed by the addition of 5 mL of concentrated nitric acid. The sample was digested at 180 °C for 25 min. After the sample cooled, the digestion tank was placed on a hot plate at 160 °C to drive out the acid, and finally, the volume was adjusted to 10 mL for use.

### 3.8. Cytotoxicity Assessment of Live Cells and In Vivo Imaging

The in vitro cytotoxicity of Y-CDs was assessed by the standard MTT assay. The 4T1 cells were placed in DMEM containing 10% fetal bovine serum and 1.0% anti-mycoplasma antibiotics and cultured in an incubator at 37 °C (5.0% CO_2_) for 24 h. Then, cells were cultured in DMEM containing various doses (0–1 mg mL^−1^) of Y-CDs for 24 h. After that, the MTT solution (10 μL, 5 mg mL^−1^) was added, which resulted in the formation of purple formazan crystals after 4 h of incubation. After the supernatant was removed, 200 μL of DMSO was added to each well and shaken in a microshaker for 10 min to dissolve the formazan crystals. Finally, optical density at 570 nm was measured by enzymatic labeling, and cytotoxicity was evaluated as follows (Equation (2)):

(2) $$cell\;viability\left(\%\right)=\frac{\left[(the\;absorbance\;of\;samples-the\;absorbance\;of\;blank)\right]}{\left(the\;absorbance\;of\;control\;group-the\;absorbance\;of\;blank\right)}\%$$

Fluorescence color photographs of freshwater shrimps were recorded with a smartphone after incubation with Y-CDs in PBS buffer solution for different times (1, 2, and 3 days) at room temperature under 365 nm UV light excitation.

## 4. Conclusions

In summary, we developed a novel portable fluorescence sensor film (Y-CDs@BCM) for rapid, selective, and sensitive detection of Cu^2+^ in pig liver, human serum, and environmental water samples. Yellow-fluorescent carbon dots (Y-CDs) synthesized from o-phenylenediamine (OPD) and 1-octyl-3-methylimidazolium tetrafluoroborate ([OMIM]BF_4_) exhibited concentration-dependent fluorescence quenching toward Cu^2+^ via the inner filter effect (IFE), achieving a detection limit (LOD) of 7.758 nM, significantly lower than previously reported methods. By incorporating bacterial cellulose (BC) as a support matrix, we mitigated aggregation-induced quenching, enhanced sensor stability, and improved portability. The Y-CDs@BCM film enabled rapid (<1 min) and visual Cu^2+^ detection using a smartphone-based analytical system, demonstrating excellent field-testing potential. Biocompatibility and bioimaging assessments via cell viability assays and in vivo imaging on freshwater shrimp confirmed the safety profile of Y-CDs. This work presents an eco-friendly strategy for trace Cu^2+^ monitoring while establishing a versatile platform for portable optical sensors in food safety and environmental applications.

## Figures and Tables

**Table 1:** Determination of Cu^2+^ in real samples (n = 3).

Sample	Added (μM)	Found (μM)	Recovery (%)	RSD (%)
pig liver	2.0	1.98	99.13	2.48
	4.0	4.57	114.34	0.77
	6.0	6.97	116.13	0.35
serum	2.0	2.01	100.73	2.74
	4.0	4.16	103.88	0.56
	6.0	5.88	98.08	1.18
environmental water	2.0	2.12	106.18	1.71
	4.0	4.05	101.33	1.34
	6.0	6.58	109.59	0.62

## Data Availability

Data are contained within the article.

## Supplementary Materials

The following supporting information can be downloaded at: https://www.mdpi.com/article/10.3390/molecules30173633/s1, Figure S1. Raman spectra of Y-CDs. Figure S2. Selectivity testing of Y-CD for various possible anions. Figure S3. Response time test of Y-CDs-Cu2+. Parker Equations (S1)–(S3). Table S1. Parameters used to calculate IFE. Table S2. Comparison of methods. Table S3. Corresponding information for all tests in this work. Refs. [34,35,36,37] are cited in Supplementary Materials.
