# Supplementary material for: Portable Bacterial Cellulose-Based Fluorescent Sensor for Rapid and Sensitive Detection of Copper in Food and Environmental Samples
Source: Molecules. 2025 Sep 5;30(17):3633. doi: 10.3390/molecules30173633 (PMC12430411; doi:10.3390/molecules30173633)
Supplement: Supplementary file 1 [file molecules-30-03633-s001.zip › molecules-3822965-supplementary.pdf]

Portable bacterial cellulose-based fluorescent sensor for rapid and sensitive detection of copper in food and environmental samples

Hongyuan Zhang [1,*], Qian Zhang [1], Xiaona Ji [1], Bing Han [1], Jieqiong Wang [2,*], Ce Han [3,*]

[1] School of Science, Changchun Institute of Technology, 395 Kuanping Road, Changchun 130012, China; 22080411109@stu.ccit.edu.cn (Q.Z.); lx_jxn@ccit.edu.cn (X.J.); hanbing626@hotmail.com (B.H.).

[2] School of Materials Science and Engineering, Changchun University, 6543, Weixing Road,

Changchun 130022, China;

[3] State Key Laboratory of Electroanalytical Chemistry, Changchun Institute of Applied Chemistry, Chinese Academy of Sciences, Changchun, 130022, China;

* Correspondence: zhanghongyuan@ccit.edu.cn (H.Z.); wangjq94@ccu.edu.cn (J.W.); hance@ciac.ac.cn (C.H.);

Procedures for handling all materials prior to testing

Transmission electron microscopy

After purification to remove salt ions and small molecule impurities, Y-CDs were dispersed in ethanol to a concentration of 0.1 mg mL^{-1}. 10 μL of the Y-CDs dispersion was then added to an ultrathin carbon film, allowed to stand for 1 minute, and excess liquid was wiped off. TEM images of the Y-CDs were then observed at 200 kV.

Fourier transform infrared spectroscopy

The purified Y-CDs were freeze-dried to a powder, and their FT-IR spectra were analyzed using the ATR method. In general, the Y-CDs powder was applied to a sample stage, and the probe was gently touched to the sample, using a crystal background for measurement. After background subtraction, 30 scans were accumulated to reduce signal-to-noise interference.

Raman spectroscopy

Due to the strong fluorescence of CDs, pretreatment is required during Raman spectroscopy. Generally, a diluted Y-CDs solution (1 mg/mL) is drawn into a quartz capillary via capillary action. A blank quartz capillary is also prepared as a background. Then, under 532 nm excitation, focusing on the central liquid column of the capillary, a blank spectrum is collected for baseline correction, followed by the collection of the Y-CDs Raman spectrum.

X-ray photoelectron spectroscopy

For solid Y-CDs testing, the purified Y-CDs were freeze-dried and then ground to prevent sticking. The samples were then coated onto a silicon wafer, purged with nitrogen, and quickly loaded. Full and high-resolution spectra of the Y-CDs were then acquired under vacuum using an Al Kα (hv = 1486.6 eV) light source.

Atomic Force Microscopy

The purified sample was dispersed in anhydrous ethanol (1 mg/mL) and sonicated for 10 minutes before being dropped onto a mica sheet and allowed to stand for 60 seconds. The sheet was then dried with nitrogen and placed in a desiccator for 20 minutes. The roughness of the sheet was measured using the tapping/AC mode.

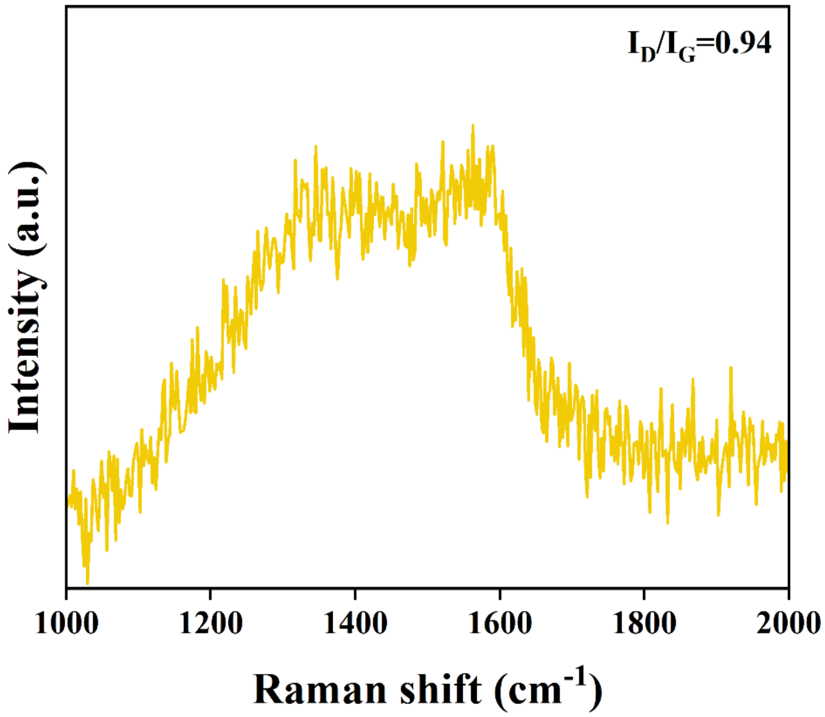

Figure S1. Raman spectra of Y-CDs.

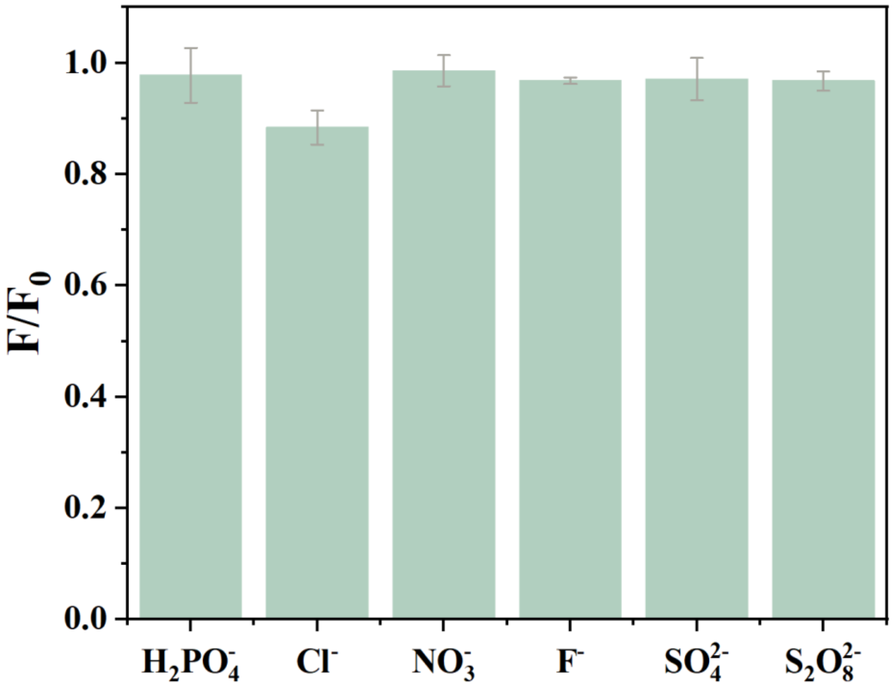

Figure S2. Selectivity testing of Y-CD for various possible anions.

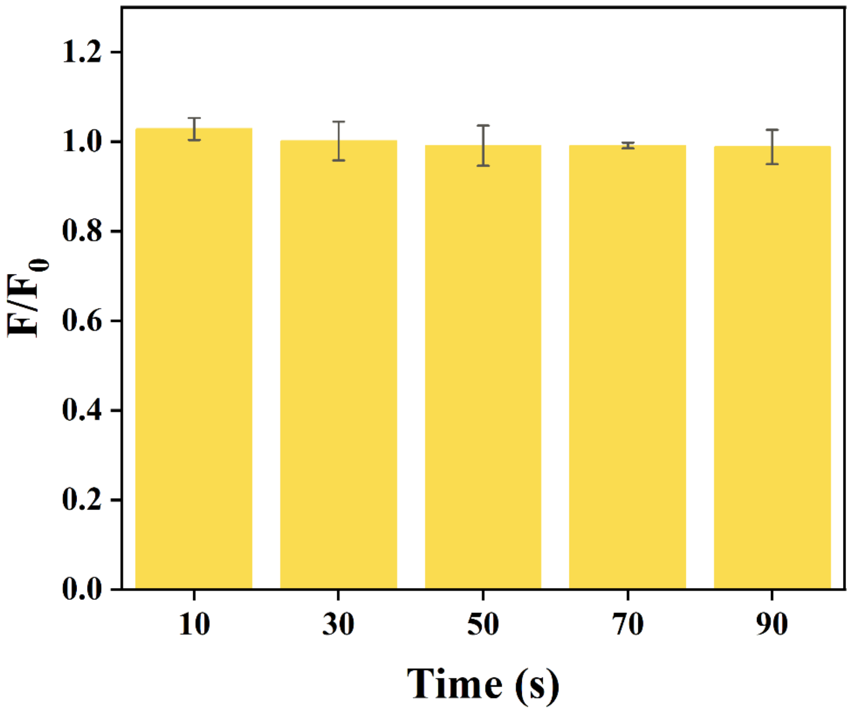

Figure S3. Response time test of Y-CDs-Cu^{2+}.

Parker equations S1, S2, and S3:

$$\frac{F_{cor}}{F_{obsd}} = \frac{2.3dA_{ex}}{1-10^{-dA_{ex}}} 10^{gA_{em}} \frac{2.3sA_{em}}{1-10^{-sA_{em}}} \quad \text{(S1)}$$

$$E_{obsd} = 1 - \frac{F_{obsd}}{F_{obsd,0}} \quad \text{(S2)}$$

$$E_{cor} = 1 - \frac{F_{cor}}{F_{cor,0}} \quad \text{(S3)}$$

F_{obsd} and F_{cor} represented the measured and corrected fluorescence intensities, respectively. The E_{obsd} and E_{cor} were the measured and corrected fluorescence intensity changes. A_{ex} and A_{em} denoted the absorbance at the optimal excitation wavelength (350 nm) and optimal emission wavelength (430 nm), respectively. "d" was the width of the cuvette and 's' was the distance between the edge of the cuvette and the edge of the excitation beam.

Table S1. Parameters used to calculate IFE

RES (μM)	A_{ex} (325 nm)	A_{em} (570 nm)	F_{cor}/F_{obsd}	E_{obsd}	E_{cor}
0.00	1.200	0.300	3.400	0	0
0.02	1.208	0.304	3.468	0.027	0.029
0.04	1.217	0.313	3.5540	0.043	0.048
0.06	1.225	0.318	3.614	0.060	0.066
0.08	1.233	0.322	3.691	0.077	0.084
0.10	1.267	0.336	4.032	0.143	0.158

Table S2 Comparison of methods.

Materials|methods	Sample	Linear range	LOD	Refs
CdSe/ZnS|FL	River water, Mineral water	0.1–200 μM	8 nM	[1]
Rhodol-TPA|FL	wine	0–1000 μM	600 nM	[2]
Carbon Quantum Dots|ECL	bovine serum	0.01–10 μM	2.78 nM	[3]
Ferrocene |DPV	Red wine	0–10 μM	29 nM	[4]
Y-CDs/Y-CD@BCM|FL	pig liver, serum, lake water	0-12 μM	7.758 nM<br>24.266 nM	This work

Table S3 Corresponding information for all tests in this work.

Sample	Quantum yield (%)	Maximum excitation wavelength (nm)	Maximum emission wavelength (nm)	Excitation and emission slit widths (nm)	Linear range (μM)	LODs (nM)	LOQs (nM)
Y-CDs	19.41 %	325	570	6	0-12	7.758	25.861

Sample	L Value	A Value	B Value	Recording equipment	Distance between equipment and sample	Linear range (μM)	LODs (nM)	LOQs (nM)
Y-CD @BCM	95	-10	25	Huawei Mate XT	15	0-20	24.266	80.887

1. Zhang, Y.-n.; Liang, W.; Li, L.; Zhang, H.; Cao, S.; Han, B.; Zhao, Y., Optical fiber fluorescence Cu2+ sensing technology based on CdSe/ZnS quantum dots: Large detection range, low detection limit. *Analytica Chimica Acta* **2024,** 1331, 343300.
2. Yang, H.; Chen, R.; Dai, L.; Ren, B.; Yang, F.; Xu, Y.-J.; Li, Q., Construction of a reaction-based fluorescent sensor for tandem detection of Cu2+ and glutathione in wine. *Food Chemistry* **2025,** 464, 141632.
3. Zhang, X. Y.; Hou, X. L.; Lu, D. C.; Chen, Y. Y.; Feng, L. Y., Porphyrin Functionalized Carbon Quantum Dots for Enhanced Electrochemiluminescence and Sensitive Detection of Cu²⁺. *MOLECULES* **2023,** 28, (3).
4. Stalin Elanchezhian, V.; Kasirajan, E.; Muthirulan, P.; Muthukrishnan, P.; Kandaswamy, M., Ferrocene-based chemosensor creates molecular logic circuit for selective detection of Hg2+ and Cu2+. *Journal of Molecular Structure* **2024,** 1313, 138687.
